# Follicular lymphoma genomics

**DOI:** 10.1097/HS9.0000000000000213

**Published:** 2019-06-30

**Authors:** Emil Kumar, Jessica Okosun

**Affiliations:** Centre for Haemato-Oncology, Barts Cancer Institute, Queen Mary University of London, London, United Kingdom

**Take home messages**

- The genetic landscape of follicular lymphoma (FL) is skewed toward frequent mutations in epigenetic regulators.
- Divergent clonal evolution from a therapy-evading common progenitor cell is proposed as the predominant mechanism underpinning relapse and transformation.
- Genomic studies are revealing new disease biomarkers and therapeutic targets, with the promise of achieving a precision medicine approach for subsets of FL patients.

## Introduction

Next-generation sequencing has improved our understanding of the genomic events that underpin follicular lymphoma (FL). In most FL tumors, the hallmark chromosomal translocation, t(14;18), co-occurs with additional genetic alterations affecting numerous biological pathways, particularly genes involved in epigenetic regulation.^1▪^^,^^2^^,^[Bibr R3][Bibr R4][Bibr R5][Bibr R6]^,^^7^ We appreciate the levels of molecular heterogeneity between tumors from different patients, but also the heterogeneity that exists within an individual as their disease evolves and progresses in space and time.[Bibr R3][Bibr R4][Bibr R5][Bibr R6],^7^ This is paralleled by our recognition of the variation in clinical phenotypes between patient populations, for example, those with localized disease versus high-risk systemic disease (such as early progressors and those who experience transformation to a high-grade lymphoma); although we have yet to fully define the molecular drivers behind such clinical behaviors. Better delineation of these, together with the molecular determinants of response and resistance to existing and emergent therapies will empower the next tranche of potential precision strategies in FL.

## Current state of the art

Genome-wide analyses now provide a comprehensive catalog of the somatic changes in FL tumors including chromosomal alterations, copy number variation, and gene mutations, the latter being the focus of this update. Recurrent gene mutations target specific biological processes, including epigenetic regulation, immune surveillance, and signaling pathways.

**Figure 1:**
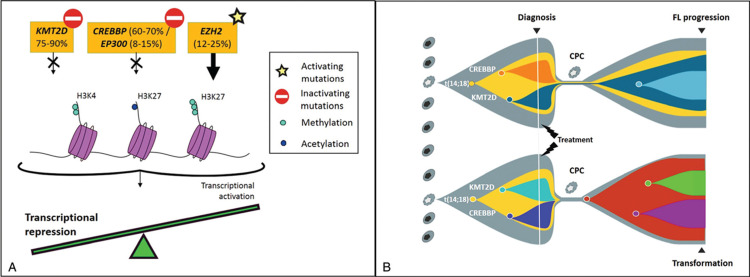
**(A) Frequently altered epigenetic modifiers in follicular lymphoma (FL) and their downstream transcriptional effects.** (B) Visualization of the clonal structure of progressed and transformed FL inferred from sequencing studies. Shown is the expansion of preexisting, therapy-resistant clones between diagnostic and progression, contrasting with dramatic clonal expansion of undetectable clones in transformed FL. All incidences arise from the common progenitor cell (CPC) harboring key genetic events.

An unexpected revelation has been the high prevalence of alterations in epigenetic regulators involved in histone post-translational modifications. Mutations in histone methyltransferases (*KMT2D*, *EZH2*) and acetyltransferases (*CREBBP*, *EP300*) are a defining feature of FL (Fig. 1).^1▪^^,^^2^^,^[Bibr R3][Bibr R4][Bibr R5][Bibr R6]^,^^7^ Almost all patients have at least one such “epimutation,”^5▪^ with most carrying multiple insults.

*KMT2D*, *CREBBP*, and *EP300* mutations are commonly inactivating, leading to loss of transcriptionally activatory marks (mono-, di-methylation of H3K4 for *KMT2D* and acetylation of H3K27 for *CREBBP* and *EP300*); whereas gain-of-function mutations in *EZH2* increase the repressive mark, H3K27 trimethylation. Functionally, these aberrations seem to exert transcriptional changes that lock cells in a germinal center (GC) stage of differentiation, while on one hand, promoting survival signaling pathways through CD40, JAK-STAT, and BCR (*KMT2D*),^8▪^ and on the other hand, perturbing immune recognition by downregulating MHC Class II expression (*CREBBP*).^5▪^^,^^9▪^

Frequent mutations affect genes involved in immune recognition (*TNFRSF14*), BCR-NFκB (*CARD11*, *TNFAIP3*), JAK-STAT (*STAT6*), and mTOR signaling (*RRAGC*, *ATP6V1B2*, *ATP6AP1*). Loss-of-function *TNFRSF14* aberrations trigger aberrant stromal activation and T follicular helper cell expansion, overall promoting a tumor-favorable microenvironment.^10^ Meanwhile, activating *RRAGC* mutations render the nutrient-sensing arm of mTORC1 signaling resistant to amino acid deprivation.^11^

Longitudinal studies have crucially delineated the clonal dynamics of progression by providing multiple snapshots of the evolving genetic repertoire during a patient's disease course. These demonstrate that relapse and transformation predominantly occur via a divergent pattern of clonal evolution: whereby all sequential tumors in a patient share a core set of mutations (Fig. 1).[Bibr R3][Bibr R4][Bibr R5][Bibr R6] This shared “trunk” of aberrations is postulated to be harbored within a putative population labeled the common progenitor cell (CPC), that can evade therapy, lay clinically quiescent over time, and act as the tumor-propagating reservoir. Importantly, these shared aberrations predominantly encompass t(14;18) together with the epigenetic mutations, affirming them as early driver events. Recently, Kridel and colleagues utilized ultra-sensitive mutation detection to describe contrasting clonal dynamics between early-relapsed FL tumors; characterized by expansion of clones already pre-existing at diagnosis, implying an inherent treatment resistance; compared with transformed FL tumors that arise from the dramatic expansion of a clone undetectable or present at extremely low levels at diagnosis.^6▪^ Unsurprisingly, the genetic drivers of transformation are heterogeneous and include alterations affecting cell cycle regulation and DNA damage response (*CDKN2A/B*, *MYC*, *TP53*), immune surveillance (*B2M*, *TNFRSF14*), and NF-κB signaling (*MYD88*, *TNFAIP3*).^3▪^,^4▪^,^6▪^ However, they are imperfect predictors for FL transformation, as many of these events also occur in untransformed FL, albeit at lower frequencies. The mutational profiles of transformed FL broadly overlap with the GC B-cell subtype of DLBCL,^4▪^ although, a minority of FL, that are predominantly t(14;18)-negative, transform to the activated B-cell (ABC) DLBCL subtype.^12^ Notably, a higher incidence of localized FL tumors lack the t(14;18) compared with advanced FL (50% cf 15%)^13^ and while t(14;18)-negative tumors share a number of typical FL-associated mutations, they also show some molecular features typical of ABC-DLBCL.^14^

The 2016 WHO revision of lymphoid neoplasm classification reflects an appreciation of the diversity of FL-related conditions,^15^ emphasized by recent genomic insights into these entities. In situ follicular neoplasia, a premalignant BCL2+ entity with low rate of progression to overt FL, has much lower genomic complexity than classical FL but already has a number of epigenetic mutations,^16^ reiterating epimutations as early events. The highly curable pediatric-type follicular lymphoma is typically t(14;18)-negative with prominent mutations affecting MAPK signaling, and a conspicuous absence of epimutations.^17^ Duodenal-type FL also follows a benign clinical course, yet bears a similar mutational profile to classical FL, although differs in its immune microenvironment gene expression signature,^18^ highlighting the significance the microenvironment niche may have in driving clinical phenotypes.

## Future perspectives

The next priorities focus on translating our increased genomic knowledge into refined diagnostic, prognostic, and therapeutic capabilities, which ultimately improve patients’ outcomes. Genomic information is beginning to be integrated into molecular-based prognostic tools that allow patients to be risk stratified at diagnosis. Molecular determinants of treatment response and resistance can serve as predictive biomarkers and are appealing as they may provide the best strategy in rationalizing how we adopt an ever-increasing armamentarium of novel therapies. This is exemplified by clinical trials examining the EZH2-inhibitor, Tazemetostat, in relapsed/refractory FL patients, with *EZH2*-mutant cases showing a superior overall response over wild-type cases.^19^ We evidently cannot rely on single-site biopsies due to the longitudinal[Bibr R3][Bibr R4][Bibr R5][Bibr R6] and spatial^7^ genetic heterogeneity in FL, and dynamic disease monitoring will be needed to overcome this hurdle. Tracking genetic signatures in circulating tumor DNA (ctDNA) could function as a multipurpose surveillance tool for monitoring tumor responses, forecasting treatment failures, and detecting disease progression.^20▪^ Application of this promising approach requires prospective validation and correlation with imaging and other biomarker strategies.

Finally, we must remember that tumor genomics represents one piece of a complex puzzle, and understanding its reciprocal interplay with aberrant epigenetic mechanisms and the tumor microenvironment will yield deeper insights into the biology.
